# Multiple Target-Specific Molecular Imaging Agents Detect Liver Cancer in a Preclinical Model

**DOI:** 10.2174/156652412802480952

**Published:** 2012-09

**Authors:** S Ke, F Zhang, W Wang, X Qiu, J Lin, AG Cameron, C Zou, X Gao, C Zau, VF Zhu, M Li

**Affiliations:** 1 Department of Radiology, Baylor College of Medicine, One Baylor Plaza, MS 360, Houston, Texas 77030, USA; 2 State Key Laboratory of Oncology in South China, Department of Imaging and Interventional Radiology, Cancer Center, Sun Yat-sen University, Guangzhou, Guangdong, P.R. 510060, China; 3 Orthopedic Surgery Center, Tang-Du Hospital, The Fourth Military Medical University, Xi’an, Shaanxi, P.R. 710038, China; 4 Department of Arteriosclerosis, Beijing Institute of Heart, Lung & Blood Vessel Diseases, Capital University of Medical Science, Affiliated Beijing An Zhen Hospital, Beijing, P.R. 100029, China; 5 Department of Biochemistry and Molecular Biology, Harbin Medical University, Harbin, Heilongjiang, P.R. 150081, China; 6 State-Province Key Laboratories of Biomedicine-Pharmaceutics of China, Harbin Medical University, Harbin, Heilongjiang, P.R. 150081, China; 7 The Vivian L. Smith Department of Neurosurgery, Department of Integrative Biology & Pharmacology, The University of Texas Health Science Center at Houston, Houston, TX 77030, USA

**Keywords:** Matrix metalloproteinase (MMP), multi-modality imaging, optical imaging, RGD.

## Abstract

Liver cancer is the fifth most common cause of cancer deaths worldwide. Noninvasive diagnosis is difficult and the disease heterogeneity reduces the accuracy of pathological assays. Improvement in diagnostic imaging of specific molecular disease markers has provided hope for accurate and early noninvasive detection of liver cancer. However, all current imaging technologies, including ultrasonography, computed tomography (CT), positron emission tomography (PET), and magnetic resonance imaging, are not specific targets for detection of liver cancer. The aim of this study was to test the feasibility of injecting a cocktail of specific molecular imaging agents to noninvasively image liver cancer. The target-specific cocktail contained agents for imaging the neovasculature (RGD peptide), matrix metalloproteinase (MMP), and glucose transport (^18^F-fluorodeoxyglucose [^18^F-FDG]). Imaging studies were performed in liver cancer cells and xenograft models. The distribution of MMP at the intracellular level was imaged by confocal microscopy. RGD, MMP, and ^18^F-FDG were imaged on tumor-bearing mice using PET, CT, X-ray, and multi-wavelength optical imaging modalities. Image data demonstrated that each agent bound to a specific disease target component. The same liver cancer xenograft contained multiple disease markers. Those disease markers were heterogenetically distributed in the same tumor nodule. The molecular imaging agents had different distributions in the whole body and inside the tumor nodule. All target-specific agents yielded high tumor-to-background ratios after injection. In conclusion, target-specific molecular imaging agents can be used to study liver cancer *in vitro *and *in vivo*. Noninvasive multimodal/multi-target-specific molecular imaging agents could provide tools to simultaneously study multiple liver cancer components.

## INTRODUCTION 

Liver cancer is the fifth most common cause of cancer deaths worldwide and its incidence is increasing [1-7]. Although early diagnosis of liver cancer by imaging is desirable, it is not always possible; 50% of patients require biopsies that entail invasive procedures [8-11]. The cellular heterogeneity of the disease reduces the accuracy of diagnostic assays. In fact, histological confirmation of early liver cancer from a limited sample remains a complex undertaking, and is often impossible [8]. Recent developments in molecular imaging permit the analysis of disease status at the level of the entire body or individual lesion, as well as at the cellular level, minimizing sampling error and permitting simultaneous analysis of multiple disease factors. 

Although nuclear medicine is the “gold standard” of molecular imaging, optical molecular imaging is a rapidly advancing imaging modality that allows simultaneous detection of multiple disease targets. Nuclear and optical imaging techniques share the same high-sensitivity detection of molecular events at nanomole to picomole levels. The combination of multi-modality imaging will provide complementary information for each individual tumor status at the molecular level. 

In the present study, we used multiple target-specific molecular imaging agents labeled with reporters to study liver cancer both *in vitro *and *in vivo*. Imaging results were validated by histopathological examination. Our data demonstrate the capabilities and value of multiple target-specific imaging in the analysis of complex disease status. These imaging results are important for understanding the heterogeneity of disease as well as for identifying interactions among critical disease components. 

## MATERIALS AND METHODS

### Cell Line

The Hepa 1-6 liver cancer cell line was purchased from the American Type Culture Collection (ATCC, Manassas, VA, USA). The cells were cultured in high-glucose Dulbecco’s Modified Eagle’s Medium/F12 nutrient medium (DMEM/F12; Invitrogen, Carlsbad, CA, USA) containing 10% fetal bovine serum (Hyclone, Logan, UT, USA) in a humidified incubator maintained at 37°C with 5% CO_2_. 

### Tumor Xenografts

Four- to six-week-old male C57L/J mice (18–22 g; Jackson Laboratory, Bar Harbor, ME, USA) were fed sterilized pellet chow (Harlan Sprague Dawley, Inc., Indianapolis, IN, USA) and sterilized water *ad libitum*. Animals were maintained in a pathogen-free mouse colony. The facility is accredited by the American Association for Laboratory Animal Care (Accredited Facility Number: 876), and all experiments were performed in accordance with the guidelines of the Institutional Animal Care and Use Committee. Tumor cells were harvested near confluence by incubating with 0.05% trypsin-EDTA. Cells were pelleted by centrifugation at 130 × *g* for 5 min and resuspended in sterile phosphate-buffered saline. Tumors were induced in animals by subcutaneous injection of approximately 2×10^6^ tumor cells into the hind leg region of each mouse. Ten mice were used in the current study.

### Target-Specific Molecular Imaging Agents 

The optical imaging agents used here were designed and synthesized as previously reported in detail [12, 13]. Briefly, the neovasculature targeting agent (RGD peptide) was labeled with Cy5.5 dye (GE Healthcare, Piscataway, NJ, USA), and the matrix metalloproteinase (MMP) agent was labeled with an NIR dye (IRDye800CW; Li-Cor Bioscience, Lincoln, NE, USA). The positron emission tomography (PET) glucose agent ^18^F-fluorodeoxyglucose (^18^F-FDG) was purchased from Cyclotope (Houston, TX, USA). 

### Confocal Microscopic Imaging 

After harvesting cultured cells, the NIR-labeled MMP agent or the free dye (100 µm) was added, and the mixture was incubated for 60 min at 37°C. The membrane dye CellTracker CM-Dil (1 µM; Invitrogen, Carlsbad, CA, USA) was added and incubated for 5 min at 37°C, and then for an additional 15 min at 4°C. Cells were then washed in phosphate-buffered saline, and Sytox Green (1 µM; Invitrogen) in 95% ethanol was added and cells were incubated for 15 min at 4°C to fix the cells and stain cell nuclei. Stained cells were then transferred to a slide and mounted for microscopic examination. Images were recorded using an Olympus confocal microscope (Fluoview 1000; Olympus America, Center Valley, PA, USA) equipped with an excitation (ex) light source and emission (em) filters to detect and separate signals from labeled MMP (ex/em, 785/810 nm), cell membrane (ex/em, 553/570 nm), and cell nuclei (ex/em, 488/510 nm). Confocal image signals were recorded from one slice of a cell z-stack. Cell nuclei, cell membrane, and NIR-labeled MMP (or NIR dye only) signals were pseudocolored green (em, 510 nm), cyan (em, 570 nm), and red (em, 810 nm), respectively. 

### Animal Imaging 

For the optical imaging study, NIR-labeled MMP (5 nmol; ex/em, 785/810 nm) and Cy5.5-labeled RGD (1 nmol; ex/em, 650/700 nm) agents were injected intravenously into tumor-bearing mice. The animals were imaged from 1 h to 2 d after injection of optical agents using a Kodak In-Vivo Multispectral System FX (Carestream Health Molecular Imaging, New Haven, CT, USA). The PET and computed tomography (CT) data were acquired 4 h after injection of 0.5 mCi of ^18^F-FDG and 0.1 ml of Omnipaque (GE Healthcare) using Siemens MicroCAT II SPECT/CT and Inveon PET systems (Siemens Medical Solutions, Malvern, PA, USA). Animals were sacrificed and dissected for organ imaging. Three-dimensional PET/ single-photon emission computerized tomography (SPECT)/CT tomographic images were co-registered by geometric transformation using Amira (Visage Imaging Inc., San Diego, CA, USA). Tumor mass, glucose uptake, as well as neovasculature and MMP signals were overlaid with anatomical images. 

### Pathological Analysis 

Tissues were fixed in 10% formalin. Paraffin-embedded tissue blocks were sectioned, and then stained using a standard hematoxylin-eosin staining procedure. 

### Statistical Analysis 

Regions of interest were quantified from *in vitro* and *in vivo* data using ImageJ (National Institutes of Health, Bethesda, MD, USA). Statistical analyses were performed using SAS software version 9.1 (SAS Institute Inc., Cary, NC, USA) for Microsoft Windows. The data were analyzed using one-way ANOVA or general linear models. Data comparisons are illustrated in notched box-and-whisker plots, which display minimum, 25th percentile, median (central line), mean (+), 75th percentile, and maximum values. The medians (central lines) of two box-and-whisker plots were significantly different at the 0.05-level (95% confidence) if the corresponding notches did not overlap. 

## RESULTS 

### Binding and Internalization of MMP Imaging Agent in Liver Cancer Cells

**Fig. (1):**
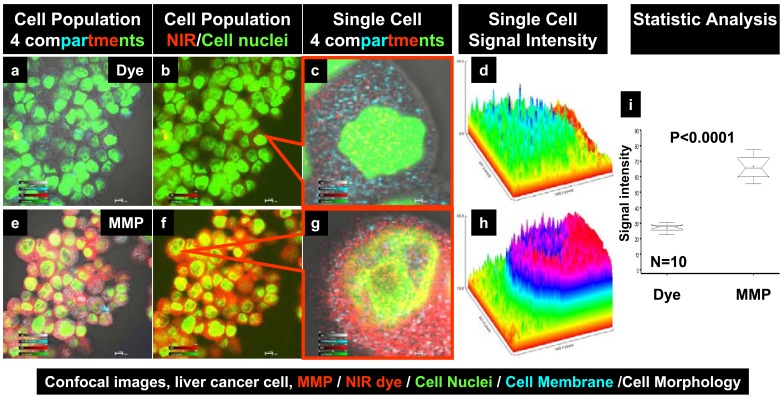
**Confocal images of MMP binding to liver cancer cells.** Cell population images show that free dye did not bind to liver cancer cells (**a–d**, red signal), whereas the NIR-labeled MMP agent bound to most liver cancer cells (**e–h**). High-magnification images of single cells showing free dye (**c**) and MMP agent (**g**) in the membrane, and cytosolic and nuclear compartments. A quantitative side-by-side comparison of agent signal intensity on the same scale (**d**, **h**). A box-and-whiskers plot demonstrates the significance of the difference in signal intensity between the MMP agent and free dye (i). (For interpretation of the references to color in this figure legend, the reader is referred to the web version of this paper).

Uptake of the NIR-labeled MMP agent into liver cancer cells was verified by NIR confocal microscopy in cell population images (Fig. **1a–h**). No detectable signal was observed when liver cancer cells were incubated with free dye (Fig. **1a–d**, red). By contrast, binding of the MMP agent to liver cancer cells was detected under the same conditions and imaging settings (Fig. **1e–h**, red). MMP signals were detected in both the cytoplasm and nucleus (Fig. **1e–g**, yellow); a higher magnification of a single-cell confocal image (Fig. **1g**) shows cytoplasmic (red) and nuclear (yellow) MMP signals. The intracellular signal intensity after incubation with the free dye (Fig. **1d**) was significantly less than that in cells incubated with the MMP agent (Fig. **1h**), as indicated by a box-and-whiskers analysis (Fig. **1i**). 

### *In Vivo* Imaging

**Fig. (2):**
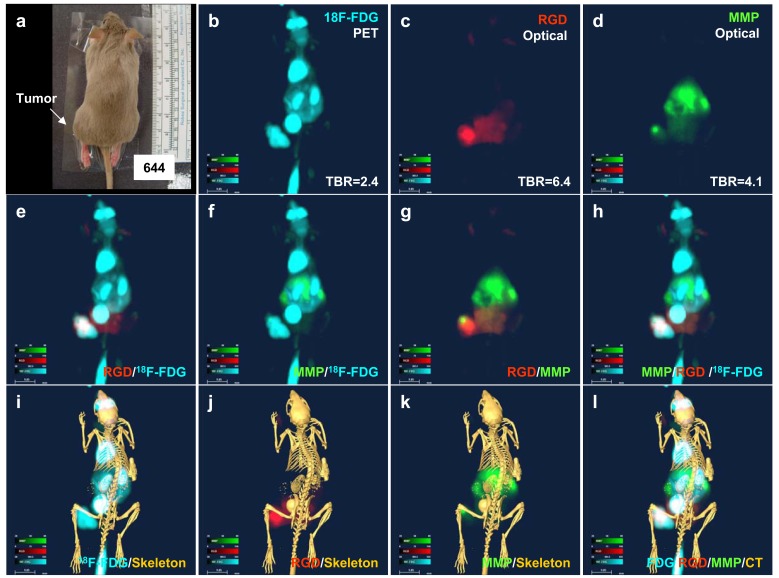
***In vivo* imaging of a liver cancer xenograft.** (**a**) The color image shows the tumor location. (**b**) ^18^F-FDG PET imaging demonstrates high glucose uptake in the tumor. (**c**) RGD optical imaging shows strong neovascular signals in the tumor. (**d**) MMP optical imaging reveals heterogeneous MMP distribution in the tumor. (**e**) Merged ^18^F-FDG PET/RGD image. (**f**) Merged ^18^F-FDG PET/MMP image. (**g**) Merged MMP/RGD image. (**h**) Merged ^18^F-FDG /MMP/RGD image. (**i**) Merged ^18^F-FDG PET/CT (skeleton) image. (**j**) Merged RGD/CT (skeleton) image. (**k**) Merged MMP/CT (skeleton) image. (**l**) Merged ^18^F-FDG/ RGD/MMP/CT (skeleton) image.

The liver tumor, located in the left hind region of the mouse (Fig. **2a**), exhibited high glucose uptake (Fig. **2b**, cyan) and showed high neovasculature (Fig. **2c**, red) and MMP (Fig. **2d**, green) signal intensities. The merged image shows the different intratumoral distribution of RGD and ^18^F-FDG signals (Fig. **2e**). Whereas most of the tumor mass was ^18^F-FDG positive, only a portion was RGD positive. The merged MMP and ^18^F-FDG image shows that a smaller part of the tumor mass was MMP positive compared with RGD (Fig. **2f**). An RGD and MMP overlay image demonstrates the completely different biodistribution of these two agents (Fig. **2g**). Most of the MMP signals were obtained from the liver, with lesser amounts in the tumor mass, whereas most of the RGD signals were from the tumor mass and the bladder. Merging MMP, RGD, and ^18^F-FDG signals into a single image (Fig. **2h**) clearly revealed tumor characteristics in detail. The results vividly show the heterogeneous distribution of the disease markers within the same tumor mass. 

To determine the origin of molecular imaging signals, we overlaid all molecular imaging results with a CT image, which confirmed the anatomical source of ^18^F-FDG signals (eyes, heart, kidneys, bladder, and tumor; Fig. **2i**), RGD signals (bladder and tumor; Fig. **2j**) and MMP signals (liver and tumor; Fig. **2k**). Fig. (**2l**) shows the difference in the whole-body distributions of the three molecular imaging agents.

### Organ Imaging

**Fig. (3):**
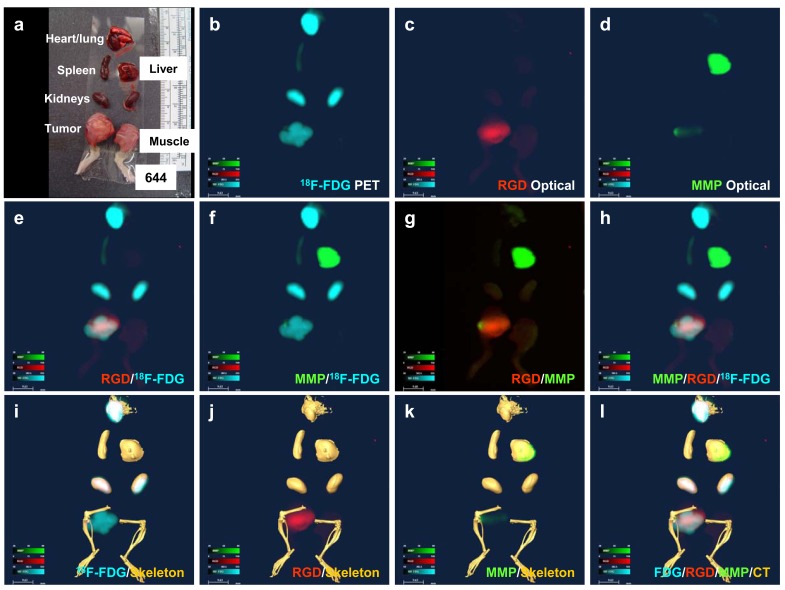
**Organ imaging of liver cancer.** (**a**) The color image shows the organ layout. (**b**) ^18^F-FDG PET imaging demonstrates high glucose uptake in different organs. (**c**) RGD optical imaging shows strong neovascular signals in the tumor. (**d**) MMP optical imaging reveals heterogeneous MMP distribution in the tumor and liver. (**e**) Merged ^18^F-FDG PET/RGD image. (**f**) Merged ^18^F-FDG PET/MMP image. (**g**) Merged MMP/RGD image. (**h**) Merged ^18^F-FDG /MMP/RGD image. (**i**) Merged ^18^F-FDG PET/CT (skeleton) image. (**j**) Merged RGD/CT (skeleton) image. (**k**) Merged MMP/CT (skeleton) image. (**l**) Merged ^18^F-FDG/ RGD/MMP/CT (skeleton) image. (For interpretation of the references to color in this figure legend, the reader is referred to the web version of this paper).

To validate *in vivo* whole-body imaging results, we dissected organs after sacrificing the animals. The organ and tissue layout is shown in Fig. (**3a**). PET imaging revealed ^18^F-FDG uptake in the heart, kidneys, and tumor (Fig. **3b**). Neovasculature imaging showed RGD signals in the tumor (Fig. **3c**), and MMP imaging showed signals originating from the liver and tumor (Fig. **3d**). A merged RGD and ^18^F-FDG organ image confirmed the *in vivo* imaging results, showing that the tumor-neovasculature distribution is different from the glucose-uptake distribution (Fig. **3e**). A merged organ image of MMP and ^18^F-FDG demonstrated that the distribution of these two agents was different and confirmed that the signal for the MMP agent was primarily in the liver and a portion of the tumor (Fig. **3f**). Consistent with the *in vivo* imaging results, a merged MMP and RGD organ image showed that most of the tumor was RGD positive and only parts were MMP positive (Fig. **3g**); furthermore, RGD background signals in non-disease organs were demonstrated to be low at this imaging time point. The overlaid ^18^F-FDG/RGD/MMP image permitted us to visualize multiple disease components at the organ level (Fig. **3h**), and injection of Omnipaque provided a view of the soft-tissue organs that usually cannot be observed under CT bone scan conditions (Fig. **3i–l**). Post-injection of CT contrast agent revealed no difference in signal intensity between tumor and normal regions (Fig. **3i–l**). Overlaying the signals of molecular imaging agents clearly defined the disease status, demonstrating that the tumor site was positive for ^18^F-FDG (Fig. **3i**), RGD (Fig. **3j**), and MMP (Fig. **3k**). The combination of anatomic and molecular imaging results provided information about the biodistribution of the different agents and allowed identification of distinct disease components (Fig. **3l**).

### Pathology

**Fig. (4):**
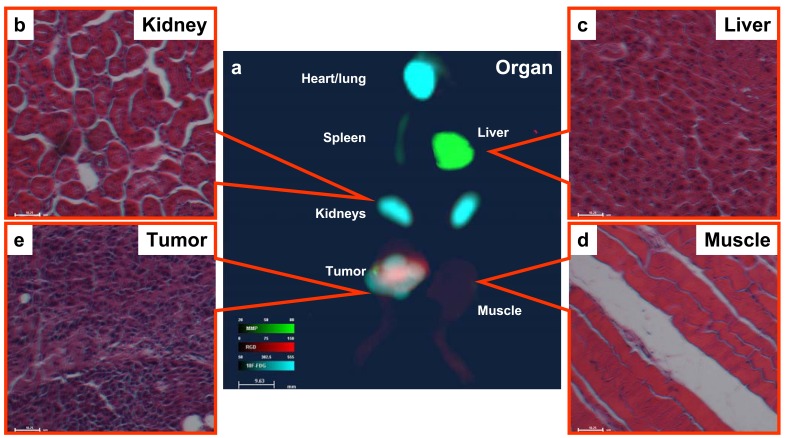
**Organ imaging and histopathology.** (**a**) Merged ^18^F-FDG /MMP/RGD image. Histological confirmation of tissue type in the kidney (**b**), liver (**c**), muscle (**d**), and tumor (**e**) using hematoxylin-eosin staining.

To verify the organ imaging results (Fig. **4a**) at the pathological level, we stained sections of kidney (Fig. **4b**), liver (Fig. **4c**), muscle (Fig. **4d**), and tumor (Fig. **4e**) tissues using hematoxylin-eosin staining. 

## DISCUSSION

Pathological analysis and immunohistochemistry (IHC) have been the standard practice for studying disease status since 1964 [14], and are the “gold standard” for disease diagnosis. The advantage of IHC is that one tissue sample can be divided into multiple samples, each of which can be tested using one or multiple antibodies with different optical reporters. Final disease status reports are based on multiple surrogate disease markers. Using surrogate disease markers reached a milestone when the United States Food and Drug Administration approved multiple surrogate molecular markers for identification of potential malignancies in ovarian cancer [15]. However, obtaining tissue biopsy samples requires invasive procedures and repeated biopsies increase the risk to the patient. Moreover, the cellular heterogeneity of the disease reduces assay accuracy. Improved molecular imaging technology provides a noninvasive approach to accurately study disease status in a longitudinal manner. Molecular imaging technology uses the same target-specific components as immunohistochemistry and permits analysis of disease status at the level of the entire body or an individual lesion, as well as at the cellular level, thereby minimizing sampling error and permitting simultaneous analysis of multiple disease factors. 

We used a liver cancer xenograft model and multiple molecular imaging agents as a test system for exploring the possibility of using a noninvasive multi-modality imaging approach to study liver cancer. Our RGD and MMP agents can bind to both murine and human disease markers [16, 17]. They are also available for dual-labeling with isotopes for nuclear imaging [16, 17]. The dual-labeled agents are optical/SPECT or optical/PET imaging agents that overcome the limitation of penetration depth typical of the optical imaging modality. 

*In vitro* cell binding studies showed that MMP bound strongly to liver cancer cells. Confocal microscopic images revealed that internalized MMP localized to the cell membrane, cytoplasm, and nucleus. MMPs are best known to be either secreted or transmembrane proteins. However, several publications have identified other intracellular localizations for several MMPs in the centrosomes and nucleus [18-20]. A side-by-side comparison and analysis under the same conditions showed that the free dye exhibited virtually undetectable binding to most cells, confirming the necessity of the targeting component of this agent. The RGD agent is targeted to the neovasculature, which only appears during tumor growth in this animal model. Therefore, RGD binding was not detected in the *in vitro* tumor cell study.

*In vivo* imaging data clearly illustrate the advantages and disadvantages of each imaging agent and modality. ^18^F-FDG/PET signals only reflect the glucose-uptake status, revealing any condition associated with high glucose metabolism, such as inflammation, exercise, or tumor. Thus, we always detected non-specific background signals in the brain, heart, kidney, and bladder with ^18^F-FDG/PET scanning because these organs either require more glucose under normal conditions or represent excretion routes. The high energy of the isotope and tomography make ^18^F-FDG/PET easy to use for whole-body scans and detection of disease deep inside the body. However, the short half-life of the isotopes and expensive detection systems limit routine use of this imaging modality as a research tool. By contrast, optical agents are stable and have a long shelf-life. Cameras are much less expensive than nuclear and CT imagers, and are easier to operate without concern about radiation exposure. The drawback of optical imaging is the limited penetration depth and the effect of animal fur on the signal, which necessitates shaving to optimize image quality. The biodistribution of each agent will also affect the study design and data collection time [21, 22]. Because liver cancer is a highly vascular tumor [23], the RGD agent only bound to the tumor and thus could potentially be used in the orthotopic liver cancer model. Conversely, the MMP agent is retained in the liver because of MMP expression in the murine liver [24]. Therefore, this agent has less opportunity to detect inoculated orthotopic or spontaneous murine liver cancer, particularly during the early stage of tumor growth. 

By analyzing pre- and post-contrast CT density, the tumor signal intensity did not increase after injection of Omnipaque. The results reinforced the common limitation of current non-target-specific diagnostic imaging technology. Most imaging agents used in clinical practice are not disease specific, particularly CT, magnetic resonance imaging, and ultrasonography contrast agents [8, 10, 25, 26]. As our knowledge of disease markers improves, particularly with increased application of DNA array and proteomic studies, multiple surrogate disease markers that provide better diagnostic information will be identified. Furthermore, the use of the multiple target-specific molecular imaging agents allows us to detect multiple disease markers according to the wavelength difference of the reporters within each imaging modality. Thus, this approach provides the promise of simultaneous identification of multiple disease components. To translate this technology into the clinic so that it ultimately benefits patients will require collaborative efforts on the part of researchers working in multiple related disciplines. 

In conclusion, we report preclinical research regarding the use of multi-target-specific agents to image liver cancer *in vitro* and *in vivo*. Using its optical properties, we studied the binding and trafficking of the MMP agent at the cellular level. Target-specific molecular imaging agents could detect a liver cancer xenograft, and the multiple noninvasive imaging agents simultaneously revealed multiple disease components in the same liver cancer xenograft. In the era of personalized and molecular medicine, this ability to identify multiple disease components will provide benefits to patients regarding early diagnosis and target-specific molecular therapy.

## ABBREVIATIONS

CT: = Computed tomography
Em: = Emission
Ex: = Excitation
FDA: = United States Food and Drug Administration
FDG or^18^F-FDG: = Fluorodeoxyglucose
IHC: = Immunohistochemistry
MMP: = Matrix metalloproteinase
NIR: = Near-infrared
PET: = Positron emission tomography
SPECT: = Single photon emission computed tomography
TBR: = Tumor-to-background ratio
